# Enhancing Musculoskeletal Injection Safety: Evaluating Checklists Generated by Artificial Intelligence and Revising the Preformed Checklist

**DOI:** 10.7759/cureus.59708

**Published:** 2024-05-05

**Authors:** Selkin Yilmaz Muluk

**Affiliations:** 1 Physical Medicine and Rehabilitation, Antalya City Hospital, Antalya, TUR

**Keywords:** chatgpt, procedure planning, artificial intelligence, patient safety checklists, musculoskeletal injections

## Abstract

Background

Musculoskeletal disorders are a significant global health issue, necessitating advanced management strategies such as intra-articular and extra-articular injections to alleviate pain, inflammation, and mobility challenges. As the adoption of these interventions by physicians grows, the importance of robust safety protocols becomes paramount. This study evaluates the effectiveness of conversational artificial intelligence (AI), particularly versions 3.5 and 4 of Chat Generative Pre-trained Transformer (ChatGPT), in creating patient safety checklists for managing musculoskeletal injections to enhance the preparation of safety documentation.

Methodology

A quantitative analysis was conducted to evaluate AI-generated safety checklists against a preformed checklist adapted from reputable medical sources. Adherence of the generated checklists to the preformed checklist was calculated and classified. The Wilcoxon signed-rank test was used to assess the performance differences between ChatGPT versions 3.5 and 4.

Results

ChatGPT-4 showed superior adherence to the preformed checklist compared to ChatGPT-3.5, with both versions classified as very good in safety protocol creation. Although no significant differences were present in the sign-in and sign-out parts of the checklists of both versions, ChatGPT-4 had significantly higher scores in the procedure planning part (p = 0.007), and its overall performance was also higher (p < 0.001). Subsequently, the preformed checklist was revised to incorporate new contributions from ChatGPT.

Conclusions

ChatGPT, especially version 4, proved effective in generating patient safety checklists for musculoskeletal injections, highlighting the potential of AI to streamline clinical practices. Further enhancements are necessary to fully meet the medical standards.

## Introduction

Musculoskeletal disorders are conditions that affect muscles, tendons, bones, cartilage, ligaments, and nerves, varying from discomfort to disabling injuries. Common ailments include low back pain, neck pain, osteoarthritis, rheumatoid arthritis, and gout. The global impact of musculoskeletal disorders is considerable, with 322.75 million new cases and 150.08 million disability-adjusted life years in 2019, highlighting their widespread prevalence and significant effect on health and quality of life [1]. Injections are valuable procedures for managing musculoskeletal conditions, relieving pain, reducing inflammation, and improving mobility. Necessary medications can be administered within the joint space (intra-articular) or around the joint space within specific soft tissue structures. They serve to definitively treat conditions, facilitate rehabilitative therapy, or provide episodic symptom relief [2]. A retrospective study of 1,188 patients showed that musculoskeletal injections significantly reduced pain, improved quality of life, and achieved high patient satisfaction, demonstrating their effectiveness in musculoskeletal problems involving knee, shoulder, and hip pain [3].

There is a surge in clinical trials for intra-articular injections, as these procedures can effectively deliver targeted therapeutics to joints and offer benefits, such as increased bioavailability, reduced systemic exposure, and lower costs, compared to systemic methods. Delivery of hyaluronate and corticosteroids accounts for the majority of intra-articular injections [4]. Despite their minimally invasive nature and relative safety, these procedures are not without risks, and although complications are rare, they can lead to unwanted situations. Adverse reactions arising from musculoskeletal injections may stem from inadequate aseptic techniques; problems with the injectate itself such as incorrect medication, utilization of a higher concentration than required, or administering an expired solution; or errors in the procedural technique such as inaccurate selection of injection sites, improper needle insertion angles, incorrect needle sizes, inadequate patient positioning, insufficient aspiration to detect blood or other fluids, and failure to adhere to proper injection depth or speed. Therefore, it is essential to implement safety measures to address these concerns. A review of complications associated with joint, tendon, and muscle injections revealed that infections are the most common adverse outcomes, including spondylodiscitis, septic arthritis, epidural abscess, necrotizing fasciitis, osteomyelitis, gas gangrene, and albicans arthritis. Others include spinal cord and peripheral nerve injuries, pneumothorax, air embolism, pain or swelling at the site of injection, chemical meningism, granulomatous inflammation of the synovium, aseptic acute arthritis, embolia cutis medicamentosa, skeletal muscle toxicity, and tendon and fascial ruptures [5]. Local infection is considered a rare complication of joint and soft tissue injections; however, it can be catastrophic and result in joint destruction [6].

The surge in clinical trials on musculoskeletal injections highlights the critical need for safety checklists to ensure the efficacy of patient care and interventions. A checklist is a structured list of tasks or criteria designed to enable a consistent evaluation by marking the completion, verification, or identification of each item. This ensures that nothing important is overlooked and promotes process standardization. Safety checklists, which were originally used in highly ordered environments such as the aviation industry, have been effectively adopted in a range of medical specialties such as surgery, hemodialysis, and anesthesiology [7]. Checklists are important tools for concisely condensing large quantities of knowledge, reducing the frequency of errors, and improving quality standards. By formulating case-specific checklists using evidence-based criteria and expert judgment, healthcare providers might be more comfortable knowing that they are providing the best-proven standard of patient care [8].

The implementation of comprehensive checklists and verification processes can improve the quality of medical practice in injection clinics. They are a critical component of moving toward excellence in patient care. Checklists such as the Surgical Safety Checklist of the World Health Organization and the Interventional Radiology Patient Safety Checklist of the Cardiovascular and Interventional Radiological Society of Europe are widely used but are specifically designed for surgical and cardiovascular procedures [9,10]. Similarly, the American College of Physicians’ Arthrocentesis and Joint Injection Checklist is available but focuses primarily on sign-in steps [11]. Therefore, there appears to be a gap in the availability of a comprehensive safety checklist for musculoskeletal injections, which should include crucial components such as procedure planning and sign-out steps besides sign-in steps.

This study investigated how conversational artificial intelligence (AI) can fill the gap in musculoskeletal injection checklists, potentially revolutionizing clinical safety and standardization. Conversational AI platforms are sophisticated systems that can comprehend and respond to a wide array of medical inquiries, offer personalized advice, and support clinical decision-making. Beyond mere text generation, they are equipped to interpret complex medical terminology, analyze patient data for insight, and potentially assist in diagnostic processes. A notable example of conversational AI is the Chat Generative Pre-trained Transformer (ChatGPT), developed by OpenAI [12]. ChatGPT is widely used in many areas, including medical research and documentation [13].

The primary objective of this study was to develop a comprehensive checklist for musculoskeletal injections based on reputable sources. The secondary objective was to assess the ability of ChatGPT versions 3.5 and 4 to generate checklists tailored to specific musculoskeletal injection interventions.

This study hypothesizes that ChatGPT-3.5 and ChatGPT-4 are capable of creating intervention-specific safety checklists that meet established medical standards with the expectation of high content integrity. It further posits that ChatGPT-4, being more advanced, will outperform ChatGPT-3.5. The null hypothesis for this study was that there would be no statistically significant difference in adherence to established medical standards between the patient safety checklists generated by ChatGPT-3.5 and ChatGPT-4 nor between these AI-generated checklists and the preformed checklist derived from reputable medical sources. Testing these hypotheses will help assess the utility of conversational AI in enhancing clinical practices and patient safety measures.

## Materials and methods

Study design

This study presents a quantitative analysis of the effectiveness of AI platforms in creating practical healthcare documents. It evaluates the ChatGPT-generated checklists by comparing each item against the adapted checklist and categorizing them from poor to excellent. Additionally, it performs a comparative performance analysis between the two versions of ChatGPT.

The Methods and Results sections of this study were prepared following the METRICS (Model, Evaluation, Timing/Transparency, Range/Randomization, Individual Factors, Count, Specificity of the prompts/language) checklist for standardization of design and reporting AI-based studies in healthcare [14]. As the study did not involve the direct participation of human subjects and was primarily focused on interactions with conversational AI systems, formal ethical approval was not sought or required.

AI model used

Two different versions of ChatGPT developed by OpenAI were selected, as they were among the most popular general-purpose conversational AI platforms during the search period. ChatGPT-3.5 was available to the public at no cost, offering users the ability to interact with an AI capable of understanding and generating human-like text based on its training data. In contrast, ChatGPT-4, the subsequent version, introduced a pricing model to access its enhanced capabilities, including an improved understanding of complex queries and the generation of more nuanced and appropriate responses.

Evaluation approach for the generated content/scoring

The content produced by the AI platforms was assessed using a checklist adapted from several authoritative sources. It mainly aligns with the European Alliance of Associations for Rheumatology’s Recommendations for Intra-Articular Therapies and the British Medical Ultrasound Society’s Guidelines for the Administration of Ultrasound-Guided Musculoskeletal Injections [15,16]. Additionally, it incorporates elements from the Cardiovascular and Interventional Radiological Society of Europe’s Interventional Radiology Patient Safety Checklist [10]. The checklist design also benefited significantly from the methodologies outlined by Hales et al. on creating clear and effective medical checklists [8]. The adapted checklist is presented in Table 1.

**Table 1:** Musculoskeletal injection checklist adapted from established guidelines and recommendations.

Procedure planning	Sign-in	Sign-out
1. Identity confirmed?	1. Patient positioned?	1. Patient observed/monitored for adverse reactions?
2. Patient informed and consented?	2. Side/site identified/marked?	2. Patient stable post-procedure (20 minutes)?
3. Allergy and sensitivity checked?	3. Asepsis confirmed?	3. Post-injection note complete?
4. Bleeding risk assessed?	4. Medication verified (name, strength, expiry)?	4. Joint activity restrictions for 24 hours advised?
5. Imaging reviewed?		5. Risks mentioned (pain, infection, flare, hyperglycemia)?
6. Contraindications checked (local, systemic, prosthetic infection)		6. Follow-up arranged?
7. Equipment checked (ultrasound, syringes, needles, solutions, gloves, etc.)?		
8. Resuscitation equipment and medication close by?		

The AI-generated checklists were scored based on the presence of items in the adapted checklist, with one point for each included item and zero points for missing items. With eight, four, and six items in the procedure planning, sign-in, and sign-out steps, respectively, the maximum possible scores were 8, 4, and 6, totaling a maximum score of 18. In addition to the raw scores, the checklists were categorized based on the total scores as follows: scores of 0-3 were considered poor, 4-7 satisfactory/fair, 8-11 good, 12-15 very good, and 16-18 excellent. Additional items present in the AI-generated checklists but not in the adapted checklist were also noted and categorized. Furthermore, the AI-generated texts were analyzed to identify any erroneous information that could potentially compromise patient safety or place the procedure at risk.

Timing of model testing/transparency of the data source

Testing of the conversational AI systems took place on February 5-7, 2024, between 08:00-10:00 Istanbul local time. The requests made to the AI and their responses are publicly accessible along with additional data via the ZENODO repository under DOI: 10.5281/zenodo.10846831.

Range of tested topic/randomization of selecting the queries

Through a systematic review of literature on Medline and Google Scholar from 2013 to 2023, using keywords “musculoskeletal injections,” “joint injections,” “tendon ligament injections,” “intra-articular injections,” and “extra-articular injections,” a comprehensive list of injection types relevant to musculoskeletal disorders was compiled. This approach ensured coverage of all significant injection sites and indications, eliminating the need for randomization.

Individual factors in selecting the requirements/count of requirements

A total of 46 specific musculoskeletal injection types were identified from the literature to be used in the prompts. To broaden the scope, two general injection types, i.e., “intra-articular injections” and “extra-articular injections,” were also included. This expansion was designed to assess the capability of AI to generate checklists for both specific and general injection scenarios. These two general types were evaluated separately from the 46 specific scenarios. The details of the injection types utilized in the prompts are presented in Table 2.

**Table 2:** Musculoskeletal injection categories that filled the gap in the standardized prompt presented to artificial intelligence.

	Body region	Injection	Condition
Specific injection categories	Shoulder region	Glenohumeral joint injection	Adhesive capsulitis
		Subacromial injection	Supraspinatus tendinitis
		Suprascapular nerve block	Shoulder pain irresponsive to conservative treatment of rotator cuff syndrome
		Acromioclavicular joint injection	Acromioclavicular joint injection
		Shoulder injection	Bicipital tendinitis
		Sternoclavicular joint injection	Sternoclavicular joint pain
		Costochondral joint injection	Tietze syndrome
	Elbow, wrist, hand	Elbow injection	Lateral epicondylitis, medial epicondylitis, olecranon bursitis
		Wrist injection	Carpal tunnel syndrome, deQuervain synovitis, ganglion cyst
		Hand injection	First carpometacarpal osteoarthritis, trigger finger, Dupuytren contracture
	Hip, pelvis, sacral region	Hip injection	Trochanteric bursitis, ischiogluteal bursitis, meralgia paresthetica, adductor tendinitis, capsulitis, psoas bursitis, iliotibial band syndrome
		Sacroiliac joint injection	Sacroiliac joint dysfunction
	Knee, ankle, foot	Knee aspiration	Knee joint effusion
		Knee injection	Gonarthrosis, Baker cyst, prepatellar bursitis, suprapatellar bursitis, patellar tendinitis, pes anserine bursitis
		Ankle injection	Tarsal tunnel syndrome, osteoarthritis, deltoid ligament strain
		Foot injection	Plantar fasciitis, Achilles tendinitis, Achilles bursitis, peroneal tendinitis, calcaneal spur, Morton neuroma, painful Bunion syndrome, metatarsalgia
	Other regions	Temporomandibular joint injection	Temporomandibular joint dysfunction
		Cervical facet joint injection	Cervical pain
		Lumbar facet joint injection	Lumbar pain
		Coccyx injection	Coccydynia
General injection categories	Intra-articular injection		
	Extra-articular injection		

No individual factors were present as all injection types were taken into account. The only intentional additions were the two general requirements for intra- and extra-articular injections.

Specificity of the prompts and language used

Each prompt followed a uniform structure, beginning with the precise introduction “I’m a physician preparing to perform...” and concluding with “I would like a comprehensive pre-procedure verification and a patient safety checklist to ensure a safe experience. Please include all relevant questions, considerations, and reminders.” This approach was designed to mimic the authentic tone of physicians seeking medical assistance. Prompt engineering or the introduction of technical terms was used according to the recommendations of Meskó [17].

To ensure replicability, both AI systems were evaluated under their standard default settings without using the “regenerate” button, and a new chat was initiated for each conversation. This study was conducted in the English language.

Statistical analysis

Statistical analyses were performed using SPSS Statistics for Windows, Version 29.0.2.0 (IBM Corp., Armonk, NY, USA). The level of statistical significance was set at p-values <0.05. To assess the adherence of the AI-generated texts to an adapted checklist for the 46 specific injection types, alignment with predefined standards was measured as a percentage, based on the total scores. Furthermore, AI performance was categorized according to the established criteria, which were also derived from the total scores. To identify potential differences between the two ChatGPT versions, the Wilcoxon signed-rank test was employed, chosen in response to non-normal distribution patterns revealed by the Shapiro-Wilk test. This comparison utilized scores from procedure planning, sign-in, sign-out, and overall scores. For the two general injection types, the analysis was restricted to descriptive statistics.

## Results

The analysis of 46 AI-generated specific injection checklists revealed that ChatGPT-3.5 had an average total score of 13.70, and ChatGPT-4 had an average total score of 14.59. Scoring was based on the predefined checklist, where each item’s presence or absence determined the score. For every item found in the AI checklist, one point was given to AI. This scoring was conducted by the researcher to ensure an objective evaluation solely based on the predefined checklist. The detailed averages for the procedure planning, sign-in, and sign-out sections of the checklist are presented in Table 3.

**Table 3:** The minimum, maximum, mean scores, and standard deviations (SDs) of 46 checklists generated by ChatGPT-3.5 and ChatGPT-4 (N = 46).

Score type	Minimum	Maximum	Mean	SD
Procedure planning score of ChatGPT-3.5	4.00	8.00	6.11	0.95
Sign-in score of ChatGPT-3.5	1.00	4.00	3.43	0.72
Sign-out score of ChatGPT-3.5	2.00	6.00	4.15	0.87
Total score of ChatGPT-3.5	11.00	16.00	13.70	1.40
Procedure planning score of ChatGPT-4	5.0	8.00	6.65	0.87
Sign-in score of ChatGPT-4	2.0	4.00	3.57	0.58
Sign-out score of ChatGPT-4	3.0	5.00	4.37	0.71
Total score of ChatGPT-4	11.0	17.00	14.59	1.20

Compared to the ideal scores of 8 for procedure planning, 4 for sign-in, and 6 for sign-out, summing to a total of 18, ChatGPT-3.5’s concordance rates were 76.38% for procedure planning, 85.75% for sign-in, and 69.17% for sign-out, achieving an overall concordance of 76.11%. ChatGPT-4 demonstrated closer alignment with the ideal, with concordance rates of 83.13% for procedure planning, 89.25% for sign-in, and 72.83% for sign-out, resulting in an overall concordance rate of 81.06%. Notably, the total score, which encapsulates the overall checklist adherence, saw ChatGPT-4 surpassing ChatGPT-3.5 by 5.2% points. When evaluating the performance of the ChatGPT versions using the scoring system detailed in the Methods section, it was found that ChatGPT-3.5 fell within the very good category, with a total score of 13.70. ChatGPT-4 was also placed in the very good category with a total score of 14.59, positioning it closer to the excellent threshold.

A comparative analysis utilizing the Wilcoxon signed-rank test of 46 AI-generated specific injection checklists revealed that ChatGPT-4 significantly outperformed ChatGPT-3.5 in the procedure planning part, with a p-value of 0.007, and in total scoring, with a p-value of less than 0.001. No significant differences were observed in the sign-in and sign-out parts, with p-values of 0.275 and 0.212, respectively. Further details can be found in Table 4.

**Table 4:** Comparative analysis of checklist scores between ChatGPT versions by Wilcoxon signed-rank test. Negative Z values suggest higher scores for ChatGPT-4. The p-values correspond to the asymptotic significance (two-tailed) and indicate the level of statistical significance for score differences.

Comparison of checklist scores	Z-value	P-value
Procedure planning (ChatGPT-4 – ChatGPT-3.5)	-2.715	0.007
Sign-in (ChatGPT-4 – ChatGPT-3.5)	-1.091	0.275
Sign-out (ChatGPT-4 – ChatGPT-3.5)	-1.249	0.212
Total (ChatGPT-4 – ChatGPT-3.5)	-3.611	<0.001

The descriptive analysis of the two AI-generated general injection checklists showed that for intra-articular injections, ChatGPT-3.5 scored 14 points (procedure planning: 6, sign-in: 4, sign-out: 4), and ChatGPT-4 scored 15 points (procedure planning: 7, sign-in: 4, sign-out: 4). For extra-articular injections, ChatGPT-3.5 achieved 12 points (procedure planning: 6, sign-in: 4, sign-out: 2), while ChatGPT-4 reached 15 points (procedure planning: 7, sign-in: 3, sign-out: 5).

In the evaluation of all ChatGPT-generated texts, the primary criterion was the identification of any erroneous information that could potentially compromise patient safety or place the procedure at risk. The analysis revealed that no content endangered patient welfare or procedural success. During the evaluation, several additional items not included in the adapted checklist were identified in the ChatGPT-generated checklists. They were about checking basal blood pressure, heart rate, respiratory rate, and temperature; asking for the pregnancy status of the patient before the procedure; and discarding used needles and syringes according to waste disposal guidelines after the procedure. The integration of these novel elements into our preformed adapted checklist framework resulted in the creation of an enhanced version of the adapted checklist. Table 5 presents the enhanced checklist.

**Table 5:** Enhanced musculoskeletal injection checklist with contributions from artificial intelligence.

Procedure planning	Sign-in	Sign-out
1. Identity confirmed?	1. Patient positioned?	1. Patient observed/monitored for adverse reactions?
2. Patient informed and consented?	2. Side/site identified/marked?	2. Patient stable post-procedure (20 minutes)?
3. Allergy and sensitivity checked?	3. Asepsis confirmed?	3. Post-injection note complete?
4. Bleeding risk assessed?	4. Medication verified (name, strength, expiry)?	4. Joint activity restrictions for 24 hours advised?
5. Imaging reviewed?		5. Risks mentioned (pain, infection, flare, hyperglycemia)?
6. Contraindications checked (local, systemic, prosthetic infection)		6. Follow-up arranged?
7. Equipment checked (ultrasound, syringes, needles, solutions, gloves, etc.)?		7. Needles and syringes discarded according to disposal guidelines?
8. Resuscitation equipment and medication close by?		
9. Basal blood pressure, heart rate, respiratory rate, and temperature checked?		
10. Pregnancy status checked?		

## Discussion

Conversational AI platforms are gaining recognition for their potential to be integrated into medical practices, offering solutions to complex healthcare challenges. This study was conducted as there was a need to harness this potential in specific clinical procedures. To our knowledge, this is the first study to evaluate the capacity of AI platforms to create checklists for musculoskeletal injections.

In this study, when the ChatGPT-3.5 and ChatGPT-4 checklists were evaluated against the adapted checklist, the concordance rates were 76.11% for ChatGPT-3.5 and 81.06% for ChatGPT-4. According to the scoring system, the checklists generated by both versions were classified as very good. The results showed that conversational AI platforms were successful in creating intervention-specific checklists for musculoskeletal injections which align with the initial hypotheses of this study.

Prior literature has underscored the potential of ChatGPT in clinical support by generating differential diagnoses, aiding in decision-making, and offering insights for cancer screening. Additionally, ChatGPT has been shown to serve as an intelligent tool for answering medical queries and enhancing medical documentation, including clinical letters, radiology reports, and discharge summaries [18]. In a case study simulation, ChatGPT analyzed a dialogue between a patient and a physician, drafted medical records, proposed differential diagnoses, and recommended treatment plans. The outcomes closely matched the physician’s summaries, indicating ChatGPT’s potential to support clinical reasoning and reduce administrative tasks, thereby freeing up more time for patient care [19]. Similarly, ChatGPT was used to prepare a clinical letter-writing process in orthopedic clinics for prior authorization approval from an insurance company. It was stated that this novel addition may save orthopedic surgeons a significant amount of time, allowing them to focus on patient care and clinical decision-making [20].

In a related study, educational materials on cirrhosis generated by chatbots matched the readability, grade level, understandability, and accuracy of materials created by humans. This led to the conclusion that educational materials produced by chatbots show promise [21]. A further investigation tasked ChatGPT to create a questionnaire on low back pain. Upon comparison with validated questionnaires, the ChatGPT-generated questionnaire revealed a notable correlation with established metrics such as the Oswestry Disability Index and Quebec Back Pain Disability Scale [22]. The outcomes of these studies are consistent with those of the present study, underscoring the role of AI in the preparation of medical materials. Furthermore, this study extends these areas by highlighting the specific utility of ChatGPT in generating safety checklists.

In the comparative analysis, ChatGPT-4 not only recorded a higher average total score but also achieved a higher concordance rate than ChatGPT-3.5. The enhancement in ChatGPT-4’s adherence to the checklist exceeded that of ChatGPT-3.5 by 5.2 percentage points. Additionally, when classified, ChatGPT-4 was closer to the excellent range. The Wilcoxon signed-rank test revealed that ChatGPT-4 significantly outperformed ChatGPT-3.5 in the procedure planning part (p = 0.007), and its overall performance was also significantly superior (p < 0.001). The improved outcomes observed with the newer version of ChatGPT also align with the hypotheses of this study, reflecting advancements in AI technology.

Likewise, a study demonstrated that ChatGPT-4 was significantly better than ChatGPT-3.5 in answering medical examination questions. ChatGPT-4 markedly surpassed ChatGPT-3.5 in performance, achieving an accuracy rate of 85.7% compared to 57.7%, and demonstrating a correctness rate of 77.8% against 44.9% in answering questions [23]. In another study, it was shown that ChatGPT-4 significantly excels ChatGPT-3.5 in accurately diagnosing and planning treatments for brain tumors, as evidenced by higher accuracy rates and positive evaluations from neurosurgeons [24]. A review reported that ChatGPT-4 outperforms ChatGPT-3.5 by leveraging enhanced training data, achieving faster processing speeds, providing more accurate answers, and showing marked improvements in language translation, question answering, and sentiment analysis tasks [25]. This evolution suggests that, as AI technologies mature, their potential utility in clinical practice will expand.

To our knowledge, this study is also the first to enhance a checklist adapted from reputable sources with additions from AI-generated texts, thereby advancing toward excellence in patient safety practices. This was done by analyzing the AI-generated checklists for additional items not included in the preformed adapted checklist. These insights from the ChatGPT versions have led to the creation of a more comprehensive safety protocol for musculoskeletal injection procedures.

However, as with any technology, there are challenges to consider when using ChatGPT. Although the integration of ChatGPT into clinical settings offers numerous advantages, there are potential disadvantages such as concerns related to privacy, ethics, bias, discrimination, and validity of the information provided [26]. ChatGPT may produce incorrect responses, and when the information provided by users is insufficient, it tends to make assumptions about what the user wants to hear [27]. Therefore, it would be ideal for users to apply appropriate prompts, as prompt engineering is essential to leverage the full potential of AI in medicine and healthcare. For example, the user should set specific requests like “What are the most common risk factors for coronary artery disease?” instead of “Tell me about heart diseases,” set realistic expectations like “What were some of the major research breakthroughs in Alzheimer’s treatment until 2021?” instead of “What’s the latest research published this month about Alzheimer?,” or use time references like “What can a patient typically expect during the six weeks of healing after knee surgery?” instead of “Describe the healing process after knee surgery” [17]. In this study, despite the use of effective prompting, the AI-generated texts fell short of achieving an excellent rating. This deficiency, when evaluated together with the mentioned concerns related to validity, underscores the necessity of expert oversight. Hence, for the safe utilization of ChatGPT in complex inquiries, a thorough review by professionals is crucial.

This study has several limitations. First, it exclusively focuses on a specific AI platform, which may narrow its scope. Second, the analysis was confined to English-language prompts, limiting its broader applicability. Additionally, while the study rigorously checked for the presence of items in the preformed checklists, it did not evaluate the clarity or practical usability of the AI-generated texts in real clinical settings, which could affect their effectiveness. The checklists have not been tested in actual clinical environments, which could provide critical insights into their practical application and effectiveness. Furthermore, this study focuses on single-joint injections and may not capture the complexities of treatments for multiple joint conditions. Future research could enhance our understanding by including a broader range of AI platforms, incorporating multiple languages, and testing these checklists in real-world clinical settings to assess their practical effectiveness and refine their design. Despite these limitations, this study represents a novel integration of AI into clinical practice and offers valuable insights into patient safety and procedural efficiency.

## Conclusions

This study demonstrates the advancements that ChatGPT brings to clinical settings, particularly in the creation of musculoskeletal injection checklists, underscoring the role of AI in enhancing patient safety and procedural efficiency. These checklists may reduce procedural errors and streamline clinical workflows, as evidenced by the improved adherence rates to best practice protocols. Despite these promising results, the study underscores the ongoing need for expert oversight. It is crucial to ensure the reliability and applicability of AI-generated content in clinical practices, confirming that AI tools function as an adjunct to, not a replacement for, human expertise.

Looking ahead, there is significant potential for expanding the application of AI in healthcare. Future work should focus on developing multilingual AI tools to improve accessibility in diverse linguistic regions and adapting AI systems for seamless integration across different healthcare platforms, ranging from electronic health records to telemedicine. The successful integration of AI into healthcare not only demands ongoing technological advancements but also a sustained collaboration among software developers, healthcare providers, and policymakers to ensure these tools are used ethically and effectively.

Ultimately, while AI can greatly augment healthcare services, it remains an adjunct tool that enhances, but does not replace, the critical judgment and expertise of healthcare professionals.
